# Cross-Breeding Is Inevitable to Conserve the Highly Inbred Population of Puffin Hunter: The Norwegian Lundehund

**DOI:** 10.1371/journal.pone.0170039

**Published:** 2017-01-20

**Authors:** Anne Kettunen, Marc Daverdin, Turid Helfjord, Peer Berg

**Affiliations:** 1 The Nordic Genetic Resource Center – NordGen, Ås, Norway; 2 NTNU Museum, Norwegian University of Science and Technology, Trondheim, Norway; 3 Norsk Hestesenter, Lena, Norway; National Cheng Kung University, TAIWAN

## Abstract

The Norwegian Lundehund is a highly endangered native dog breed. Low fertility and high frequency predisposition to intestinal disorder imply inbreeding depression. We assessed the genetic diversity of the Lundehund population from pedigree data and evaluated the potential of optimal contribution selection and cross-breeding in the long-term management of the Lundehund population. The current Norwegian Lundehund population is highly inbred and has lost 38.8% of the genetic diversity in the base population. Effective population size estimates varied between 13 and 82 depending on the method used. Optimal contribution selection alone facilitates no improvement in the current situation in the Lundehund due to the extremely high relatedness of the whole population. Addition of (replacement with) 10 breeding candidates of foreign breed to 30 Lundehund breeders reduced the parental additive genetic relationship by 40–42% (48–53%). Immediate actions are needed to increase the genetic diversity in the current Lundehund population. The only option to secure the conservation of this rare breed is to introduce individuals from foreign breeds as breeding candidates.

## Introduction

The Norwegian Lundehund is a highly endangered dog breed, native to Norway [1], listed as a national Norwegian dog breed. The name of the Norwegian Lundehund originates from its function to retrieve puffins alive (*Fratercula arctica*, lunde in Norwegian) from their nests in screes and burrows on steep mountainsides along the Northern Norwegian coast [1,2]. There is evidence that this hunting method dates back as far as to the 16^th^ century [1]. Lundehund were highly appreciated and secured both income (down feather) and food for families living in fisherman’s villages near seabird colonies [1]. In the 1850s hunting with nets became popular and the need for Lundehund disappeared. Consequently, the number of Lundehund reduced drastically. At the outbreak of the Second World War the population size was 50 individuals [1]. Since then the Lundehund population experienced two severe bottlenecks. As a result the current Lundehund population stems from only a very few related individuals.

It is expected that the bottlenecks have caused a great loss of genetic diversity in the Lundehund population. Visual evidence for this loss of diversity is the reduced variation of coat colours. Before the Second World War black and white Lundehund existed, whereas at present these variants are extinct [1]. Reduced litter size has been suggested as a sign of inbreeding depression in the Lundehund. Currently, the Norwegian Lundehund club reports average litter sizes of 2.8. Low average litter size is a reflection of a high proportion of one-puppy litters and near complete absence of litters with six or more puppies [3]. Moreover, breeders have reported problems relative to fertility, such as “invisible” heat, behavioural problems when mating, both in males and females, and low sperm quality. Overall, reduced litter size most likely reflects inbreeding depression in several traits, both physiological and behavioural, as well as a high frequency of non-viable embryos due to lethal alleles.

In the 1960s breeders discovered that many dogs were suffering from intestinal problems, later diagnosed as intestinal lymphangiectasia (IL) [1]. Untreated IL is a life threatening disease, but even with an early diagnosis and veterinary treatment the survivors risk a relapse. About 30% of the reported causes of death in the Lundehund in Norway are due to IL [3]. Due to the complicated disease mechanisms and unknown mode of inheritance [4], breeding efforts have failed to reduce the high frequency of this disease in Lundehund [1]. It may be speculated that such a high disease frequency is also a consequence of inbreeding. Pfahler and Distl [5] suggested that selection for body size or male fertility might have predisposed the Lundehund to gastroenteropathies.

Molecular genetic studies have verified extremely low genetic diversity in the Lundehund. Melis *et al*. [2] reported observed heterozygosity of 0.075 for 26 microsatellite loci in a Norwegian sample of Lundehund. Based on SNP markers (single nucleotide polymorphism), Pfahler and Distl [6] found an observed heterozygosity of 0.047 and high F_IS_ (0.87) indicating close relatedness between the individuals. In comparison, the heterozygosity in five Danish dog breeds was estimated to be between 0.27 and 0.36 [7]. Similarly, a recent study verified significantly lower genetic diversity in the Lundehund than in three other Nordic Spitz breeds: Norwegian Buhund, Icelandic sheepdog and Norrbottenspets (unpublished data, Stronen A).

The Norwegian Lundehund club, the organization responsible for breeding and conservation of the Lundehund, has initiated a cross-breeding project to improve health and welfare of the population. In particular, it is critical to reduce the risk of developing IL and improve fertility without compromising the preservation of the distinctive characteristics of the breed. The Norwegian Kennel Club, the head organization for breed organizations, has granted permission to cross three phenotypically similar breeds into the Lundehund: Norwegian Buhund, Icelandic sheepdog and Norrbottenspets. To date, only a few attempts of crossbreeding have been made, and only two of those matings have resulted in litters.

The sustainable management of small populations is dependent on the long term control of increases in additive genetic relationships. Optimal contribution selection (OCS) is a method that allows maximisation of genetic merit of a cohort of animals, while setting constraints on the average relationships between the individuals. OCS has proven to be a robust method also in complex breeding scenarios, imposed by various restrictions, realising most of the long-term genetic gain obtained by OCS without restrictions [8]. Many small native populations have a pure conservation focus, and consequently the genetic contributions of selection candidates, individuals available for breeding, are optimised only based on the additive genetic relationships.

Our overall goal was to evaluate the conservation possibilities of the current Lundehund population. Consequently, we assessed the genetic diversity of the Lundehund population from pedigree data in terms of levels of inbreeding and relationships, probability of gene origin, and effective population size. We furthermore evaluated the potential of OCS and cross-breeding in the long-term management of the Lundehund population.

## Materials and Methods

### Data

Pedigree data from 1930 to 2015 comprising records of 5433 individuals was obtained from the Norwegian Lundehund Club. The data included identification of the individual (number and name), identification number of sire and dam, country of origin, country of residence, sex, birth date, information whether the dog is alive or not, and number of offspring. A total of 1220 individuals were registered as alive, of which 50.4% were living in Norway. The largest populations of Lundehund outside Norway are situated in Sweden (N = 156), Finland (N = 106) and Denmark (N = 78). The distribution of the current global Lundehund population is presented in Table 1. For OCS analyses dogs born between 2007 and 2014 were considered as breeding candidates. To avoid that parent(s) and offspring were allocated to the same reproductive cohort the contemporary group (later referred to as a time step) was defined as a six-month period within each birth year, e.g. 2014/1 (January-June) and 2014/2 (July-December).

**Table 1:** Distribution of the current global Lundehund population.

Country	Number of dogs	%
Norway	617	50.41
Sweden	156	12.75
Finland	106	8.66
Denmark	78	6.37
The Netherlands	67	5.47
Germany	55	4.49
USA	49	4.00
Switzerland	30	2.45
France	18	1.47
Czech Republic	17	1.39
Austria	8	0.65
Luxembourg	7	0.57
Belgium	3	0.25
Iceland	2	0.16
Cyprus	2	0.16
Poland	2	0.16
Croatia	1	0.08
Lithuania	1	0.08
Spain	1	0.08
Total	1220	100

The current pedigree dates back to the 1930s and these founder dogs were considered as unrelated. If the dogs used to construct the current population were related, the level of relatedness and average inbreeding in the current population will be underestimated.

### Statistical analyses

Population analyses relative to inbreeding, additive genetic relationships and pedigree completeness were carried out using EVA 2.1 [8,9]. The effective population sizes were estimated by ENDOG v.4.8 [10]. ENDOG utilizes, by default, either the full (F) or restricted (R) pedigree data set, where individuals with unknown parentage and generation coefficient < 2.0 were discarded from a restricted pedigree data set, in order to take into account that ancestral information has to be known for a minimum of two generations back to detect inbreeding. Individuals with a low level of pedigree information would contribute to overestimation of N_e_. The effective population size based on different definitions of Δ*F* was computed as $N_{e}=\frac{1}{2\Delta F}$.

First, three simple regression based methods were used for estimating Δ*F* from the full data set (Table 2). Individual inbreeding coefficients were regressed on the number of complete generations (method I a), the maximum number of generations (method I b) and the number of equivalent generations traced back for each individual (method I c) and the regression coefficient (*b*) was used as the increase in inbreeding between two generations. Consequently, N_e_ was estimated as $N_{e}=\frac{1}{2b}$ [10]. The regression coefficient (*b*) of the individual inbreeding coefficients over the equivalent generations was used to approximate $\Delta F\approx \frac{b}{1-\left(F_{t}-b\right)}$ in method II. In the third regression based method (method III), following Gutiérrez *et al*. [11], the inbreeding coefficients are regressed on birth years and the increase in inbreeding between two generations is defined as the product of regression coefficient (*b*) and the average generation interval (*l*): *l* × *b*. From this, $\Delta F=\frac{l\times b}{1-\left(F_{t}-\left(l\times b\right)\right)}$, where *F*_*t*_ is the average inbreeding of the population in question. Additionally, values of log(1 − *F*_*i*_) were regressed on birth years (method IV a) or complete generation equivalents (method IV b) (Pérez-Enciso 1995) and $\Delta F=\frac{\left(1-e^{b}\right)}{l}$ was calculated.

**Table 2:** Summary of the methods used for estimation of effective population size.

Method	Definition of Δ*F*	Regression on	Pedigree data set
I a	*b*	Number of complete generations	F
I b	*b*	Maximum number of generations	F
I c	*b*	Complete generation equivalents	F
II	$\frac{b}{1-\left(F_{t}-b\right)}$	Complete generation equivalents	R
III	$\frac{l\times b}{1-\left(F_{t}-\left(l\times b\right)\right)}$	Birth years	F
IV a	$\frac{\left(1-e^{b}\right)}{l}$	Birth years	F
IV b	$\frac{\left(1-e^{b}\right)}{l}$	Complete generation equivalents	R
V	$\Delta F_{i}=1-\sqrt[{t-1}]{1-F_{i}}$	Not relevant	R
VI	$\Delta C_{ij}=1-\sqrt[{\left(t_{i}+t_{j}\right)/2}]{1-C_{ij}}$	Not relevant	R

F = full pedigree data set; R = restricted pedigree data set with individuals with unknown parentage and generation coefficient (complete generation equivalent) < 2.0 discarded; V and VI definition is for individual increase in inbreeding and co-ancestry, respectively. Full description of methods III, IV b, V and VI are found in [11], [12], [13,14] and [15], respectively.

In method V, N_e_ via individual increases in inbreeding (Δ*F*_*i*_) was estimated according to Gutiérrez *et al*.[13]. “Realized effective size” was computed as $\bar{N_{e}}=\frac{1}{2\bar{\Delta F}}$, where $\bar{\Delta F}$ is computed as ΔF¯=(∑iN1−1−Fit−1)N, where *F*_*i*_ is the individual inbreeding coefficient, *t* the generation coefficient of individual *i*, and N is number of individuals in the reference population [13,14]. The standard error of $\bar{N_{e}}$ can be computed as: $\sigma_{\bar{N_{e}}}=2{\bar{N_{e}}}^{2}\sigma_{\bar{\Delta F}}\frac{1}{\sqrt{N_{e}}}$ [14]. Finally, a method based on the increase in co-ancestry for all pairs of individuals in the reference population was used for the estimation of N_e_ (method VI). Averaging out the increase in co-ancestry in all pairs of individuals in the reference population provides an estimate of $\bar{\Delta c}$ and $\bar{N_{ec}}=\frac{1}{2\bar{\Delta c}}$. The standard error of the estimate $\bar{N_{ec}}$ is calculated in a similar manner as in the method of Gutiérrez *et al*. [13,14]. See, Cervantes *et al*. [15] for a complete description of this method.

Parameters derived from the probabilities of gene origin were used as complementary to increase in inbreeding and effective population size to describe genetic diversity in the Lundehund population. The effective number of founders or the founder equivalent (*f*_*e*_) is defined as the number of equally contributing founders that would be expected to result in the same genetic diversity as in the population under study, thus accounting for the loss in genetic variability due to unequal founder contributions [16]. It is calculated as an inverse of the sum of the squared proportional contributions (*p*_*i*_) of the founders fe=1∑p=1fpi2. The effective number of ancestors (*f*_*a*_) is defined as the minimum number of ancestors (not necessarily founders) explaining the complete genetic diversity of the population. Parameter *f*_*a*_ is calculated as *f*_*e*_ but replacing *p*_*i*_ with marginal ancestral contributions (*m*_*i*_), and thus unlike *f*_*e*_, accounts for potential bottlenecks in the pedigree [17]. Finally, the founder genome equivalent *f*_*ge*_ [16,18] takes into account the number of founder alleles already lost from the population both due to bottlenecks and genetic drift. This parameter accounts for all causes of gene loss and describes how many alleles and in which frequencies they have been maintained in a given locus [17]. It may be approximated using both the proportion of genes contributed by a given founder in the descendant population and the expected proportion of alleles retained within the descendant population [18]. Lacy [18] introduced the exact definition of *f*_*ge*_ which may be obtained from the average co-ancestry of a predefined reference population *t* as fge=12α¯ [19]. The effective number of non-founders was calculated as $f_{ne}=\;{\left[\frac{1}{f_{ge}}-\frac{1}{f_{e}}\right]}^{-1}$, measuring the amount of genetic drift since the foundation of the population. The parameters *f*_*e*_, *f*_*ge*_ and *f*_*ne*_ were used to derive measures of genetic diversity. The degree of genetic diversity relative to the base accounting for loss of diversity due to unequal founder contribution was calculated as $GD*=1-\frac{1}{2f_{e}}$ [18] and accounting for unequal founder contribution, bottlenecks and genetic drift as $GD=1-\frac{1}{2f_{ge}}=1-\bar{\alpha }$ [18,19]. The difference $GD*-GD=\frac{1}{2f_{ne}}$ measures the loss in diversity due to drift accumulated over non-founder generations. Using the above expressions, the proportional loss of diversity due to random genetic drift was simplified to $1-\frac{f_{ge}}{f_{e}}=1-\frac{1}{2\bar{\alpha }f_{e}}$. Consequently, proportional loss due to unequal founder contribution is $\frac{f_{ge}}{f_{e}}$. Reference population in the estimation of parameters based on the probabilities of gene origin was defined as all living individuals (Table 1).

The software package ENDOG was used to obtain the N_e_ estimates, but all, except that of Cervantes *et al*. [15], are easily calculated from the general output files of both EVA and ENDOG following the formulas given above and the full descriptions of the methods in the literature [11,13–15]. Parameters *f*_*e*_ and *f*_*a*_ were obtained from ENDOG whereas *f*_*ge*_ was calculated as explained above from EVA output files.

EVA 2.1 software was used for the OCS analyses [8,9]. To assess the possible effect of importation on the management of the Norwegian Lundehund population, three different data sets were analysed: global, Nordic and Norway (Table 3). Additionally, following the recommendations of the Norwegian Lundehund club prohibiting the use of cross-bred individuals in breeding at the moment, an additional analysis excluding cross-bred individuals was carried out. Three scenarios of limited use of males within each data set were tested, allowing males to be used in 1, 5 or 10 matings. A total of 20 matings were optimized in the OCS analyses.

**Table 3:** Number of male and female candidates and the relatedness between male and female candidates in the different OCS scenarios.

	Males	Females	Relatedness*
Global	462	478	0.77
Nordic	365	362	0.77
Norway	237	224	0.77
Norway, no-crossbreds	235	220	0.78

* average genetic relationship between male and female candidates including relationship to itself (1+F_i_).

## Results and Discussion

### Pedigree completeness

**Fig 1:**
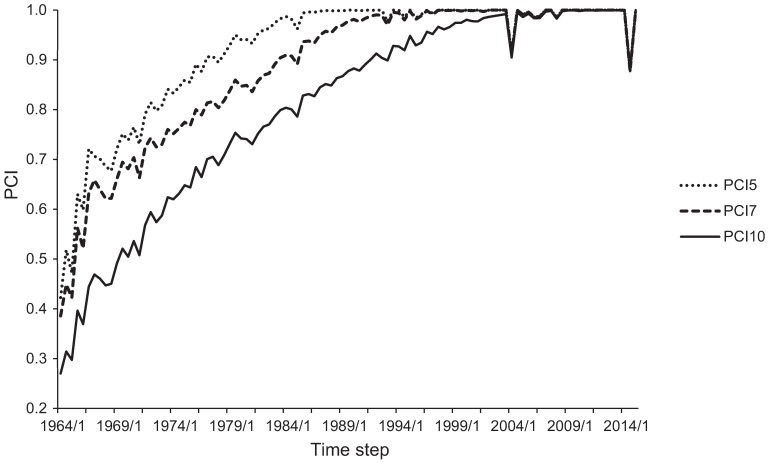
Pedigree completeness (PCI) between 1964–2015 for pedigree completeness indices of 5 (PCI5), 7 (PCI7) and 10 (PCI10) generations. Time steps are defined as 6-month periods within a year: /1 = January-June, /2 = July-December.

Pedigree completeness [20] of the Lundehund from the start of 1964 is presented in Fig 1. The most conservative pedigree completeness index (PCI), looking back 10 generations (PCI10), reached 90% completeness during the early 1990s (Fig 1). The same level of PCI was reached in the early 1980s and late 1970s for PCI7 and PCI5, respectively. During the same time period, the depth of the pedigree increased from 6 to 19 generations.

The drops in PCI in 2004 and 2014 are due to the proportion of unknown parentage: 9% (2004) and 12% (2014). Two incidences of drops in the average level of inbreeding coincide with these declines in PCI10. The first case is a result of “unknown” parentage of an imported individual causing an underestimation of the level of inbreeding in that cohort, as all Lundehund originate from Norway and thus are related to each other. The second case, in 2014, is a real fall in inbreeding as a consequence of using a Norwegian Buhund dam in breeding. High PCI of the Lundehund pedigree data ensures reliable estimation of the average levels of inbreeding in the current population.

### Genetic contribution of founders and ancestors

There were 49 founder animals (*f*) in the data set. If an animal with one unknown parent was defined as a half founder, the actual base population consisted of 43.5 individuals. Out of these, only 11 contributed to the last cohort of 2015 (percentage of genetic contribution in parentheses). Eight of the founders (7.0%) are parents born in the 1930s, each having one contributing offspring. One male founder (4.7%) has three offspring born in the 1960s, of which two have further contributed to the last cohort. The founder male contributing most to the last cohort has 10 offspring all born in the 1960s, which all have further contributed to the population. This individual is the second most contributing ancestor in the pedigree, with 35% contribution to the last cohort.

The ancestor with the highest contribution in the pedigree is a female with 18 offspring born in the 1960s. Her contribution to the last cohort is 41%. Her pedigree can be traced back to the base population through three rounds of full-sib mating, making her 50% inbred. The cumulative proportion of genetic variation explained by these two most contributing ancestors is 76%. In other words, on average 76% of alleles in individuals born in the first half of 2015 originate from these two ancestors.

### Relatedness and levels and trends of inbreeding

**Fig 2:**
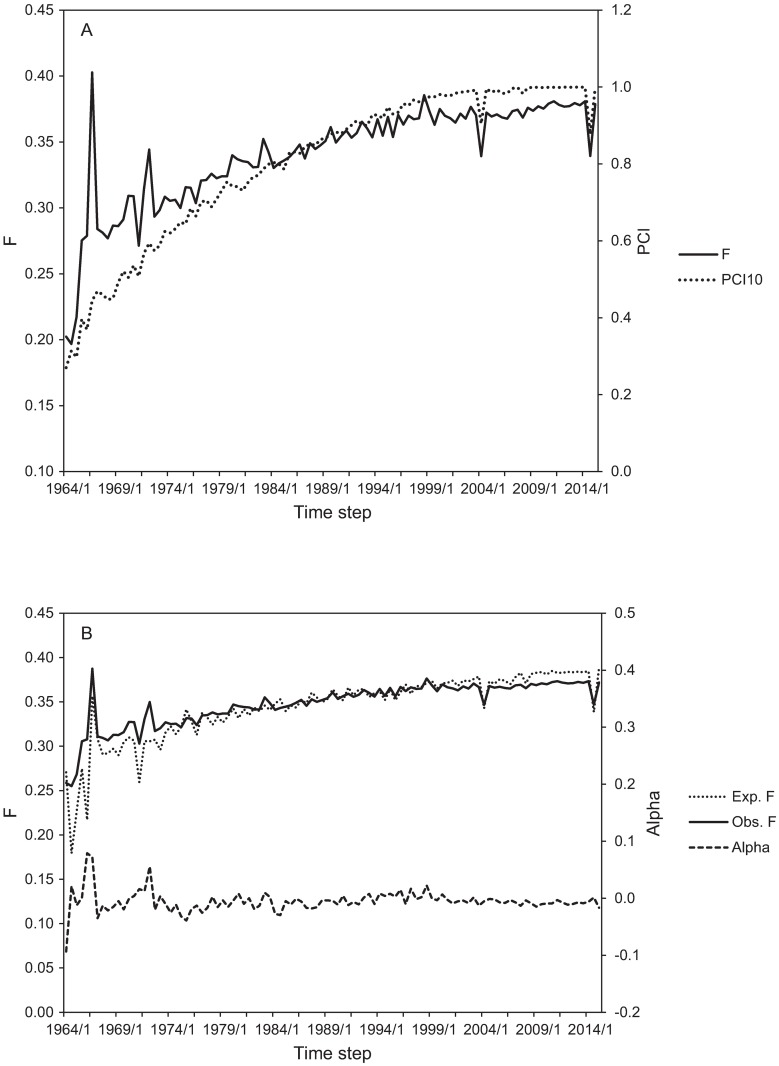
Average inbreeding and pedigree completeness in the Lundehund population between time steps 1964/1 and 2015/1. (A) Average inbreeding (F) and pedigree completeness considering 10 generations (PCI). (B) Expected and observed inbreeding and their deviation (alpha). Time steps are defined as 6-month periods within a year: /1 = January-June, /2 = July-December.

The average co-ancestry and inbreeding coefficient in the last fully registered cohort (2014/2) was 35.6% and 33.9%, respectively. The development of the level of inbreeding (F) between 1964/1 and 2015/1 together with PCI10 is shown in Fig 2, panel A. The estimated rate of inbreeding (Δ*F*) throughout the pedigree is 1.0% per generation if linear regression of F over time was used for estimation. $\;\bar{\Delta F}$ based on the individual increases in inbreeding was 4% [13].

In total, 33 individuals registered in the pedigree had an inbreeding coefficient equal to or larger than 0.5. One generation of self-fertilisation or three generations of full-sib mating result in inbreeding coefficients of 0.5. Of the total number of matings, 0.98% (N = 53), 2.04% (N = 111) and 0.81% (N = 44) were full-sib, half-sib or parent-offspring matings, respectively.

The global Lundehund population is highly inbred. To our knowledge, this is the second highest level of inbreeding reported for dogs in the scientific literature. Since 1996 the average within cohort inbreeding has been relatively stable between 36–38%, with the exception of two cohorts (2004/1; 2014/2), where importation and the use of a foreign breed lowered the average inbreeding to 34% (Fig 2, panel A). In the Lundehund, the expected and observed levels of inbreeding have not significantly deviated since the early 1970s (alpha in in Fig 2, panel B). This implies that the intended avoidance of mating between close relatives have had no effect on the levels of inbreeding as the co-ancestry between all breeding candidates is uniform.

Several studies have reported average inbreeding coefficients for various dog breeds either assessed by genealogical or molecular data. Only one pedigree-based estimate of inbreeding for Lundehund has been reported prior to our study; Pfahler and Distl [5] have recently estimated inbreeding in Lundehund from pedigree data reaching back 11 generations. Their estimate of mean inbreeding of 10% is likely an underestimate as there was no knowledge on relatedness of the founders of the pedigree. Typically, low or moderate pedigree-based average inbreeding coefficients has been published in the scientific literature for numerous dog breeds (a selection summarized in Table 4). Only a few studies have reported levels of average inbreeding comparable to those found in this study (0.21–0.45). In some cases, level of inbreeding is likely to be underestimated due to a shallow or sparse pedigree.

**Table 4:** Average inbreeding coefficients in various dog breeds, a selection.

Breed	Mean F	Reference
Rough Collie	0.073	[21]
Labrador Retriever	0.024	[21]
Greyhound	0.058	[21]
Polish Hound	0.37	[22]
“Healthy” breeds	0.045*	[23]
“Unhealthy” breeds	0.025*	[23]
Norrbottenspets	0.047	[23]
Norwegian Buhund	0.054	[23]
The Pyrenean Mountain Dog	0.04	[24]
Barbet	0.124	[24]
The Pyrenean Shepherd	0.088	[25]
The Czechoslovakian Wolfdog	0.002	[25]
Great Dane	0.044	[25]
Nova Scotia Duck Tolling Retreiver	0.26	[26]
Lancashire Heeler	0.10	[26]
Icelandic Sheepdog	0.21	[27]
Bouvier des Ardennes	0.45	[28]

*dogs alive at the end of 2010, mean of healthy (N = 11) or unhealthy breeds (N = 16)

### Effective population size

Table 5 summarizes the results of the estimation of N_e_ with different methods. Regression based methods estimated up to over 6-fold N_e_ (34–82) compared to estimates based on individual increase in inbreeding or co-ancestry in the Lundehund (13) [13,14]. As long as the full pedigree information is utilised, methods accounting for full pedigree history were relatively invariant from the choice of the sample used for N_e_ estimation, whereas the regression on birth date was very sensitive to the choice of the time period (Table 6). When regression based estimates were obtained using a time period with relatively stable level of inbreeding, overestimation of N_e_ was drastic (Fig 2, Table 6).

**Table 5:** Estimates of effective population size (N_e_) estimated with different methods. For methods V and VI, standard error in parentheses.

	Method	N_e_
**Whole pedigree**		
I a:	Regression on complete generations	34
I b:	Regression on maximum generations	82
I c:	Regression on equivalent generations	47
III:	Regression on birth date	33
IV a:	Log regression on birth date	37
**Restricted pedigree**		
II:	Regression on equivalent generations	38
IV b:	Log regression on equivalent generations	41
V:	Individual increase in inbreeding	13 (1.35)
VI:	Individual increase in co-ancestry	13 (0.45)

**Table 6:** Effect of the choice of reference population on the estimation of effective population size (N_e_).

	N_e_	
Time period	Individual increase in inbreeding	Regression on bir date
1964–2015	13	47
1930–1972	8	13
1973–1999	11	34
2000–2015	16	134

Individual increase in inbreeding refers to the method of Gutiérrez *et al*. [12,13] and regression on birth date to that of Gutiérrez *et al*. [11].

N_e_ limits are often used as indicators for sustainability and endangerment. Populations with lower N_e_ than 100 are said likely to be at risk for substantial losses in genetic diversity, and those with an N_e_ lower than 50 at high risk of detrimental effects of inbreeding [29]. No earlier pedigree based estimates of N_e_ are available for Lundehund. The present study estimates the N_e_ of the global Lundehund population to be very low (13). Based on the number of breeding animals, the effective population size for the Lundehund has earlier been reported to be approximately 80 [3]. Pfahler and Distl [5] estimated inbreeding and N_e_ from a molecular data of 28 individuals of Lundehund. Their estimate for the last 7–200 generations was 10–13, in good agreement with our pedigree based estimate, supporting a genetically small and closed population [5].

Regression based and highly variable estimates of N_e_ have been reported for a large group of dog breeds [21,23–26,29]. Leroy *et al*. [24] reported realized effective population sizes of 20–147 in nine French dog breeds, and in a more recent paper an N_e_ of 46–2136 in 61 dog breeds in France [25]. According to Calboli *et al*. [21] N_e_ varied between 17 (Greyhound) and 114 (Labrador retriever) in10 dog breeds registered in the UK Kennel Club since 1970. In a more recent paper, Lewis *et al*. [29] reported trends in genetic diversity in all dog breeds currently recognized by the UK Kennel Club. Most likely the estimates therein are overestimates, as the time span of this research is 1980–2014 and relatedness before 1980 was not accounted for. A recent study by Wijnrocx *et al*. [28] reported highly variable N_e_ estimates based on both individual increase in inbreeding (3.2–829.1) and individual increase in co-ancestry (4.5–334.9) in total of 23 Belgian dog breeds. The variability in the reported estimates of N_e_ arises from the breed history, the amount and quality of the pedigree information, and in particular, from the method used to obtain the estimates. In regression based methods, inbreeding coefficients are regressed on either generations or year of birth. Consequently, the choice of the time period used for calculation of N_e_ becomes decisive: flat or negative trends of inbreeding will result in illogical estimates of N_e_ [14]. As long as the full pedigree information is utilised methods based on individual increases in inbreeding/co-ancestry describe the breed history, inclusive effects such as mating policy, drift and selection. Thus, these methods are expected to result in estimates invariant from the choice of the sample used for N_e_ calculation [14]. Additionally, this approach allows ascertainment of the confidence in the estimate, because the corresponding standard error of the estimate is easily computed.

### Probability of gene origin

Parameters of probability of gene origin were estimated to be 6, 3, 1.3 and 1.6 for *f*_*e*_, *f*_*a*_, *f*_*ge*_ and *f*_*ne*_. Only a small proportion of the base population contributed to the current Lundehund population, reflected by low ratio of *f*_*e*_ to *f* (0.12). Comparison of *f*_*e*_ and *f*_*a*_ in Lundehund indicates substantial loss of genetic variation due to bottlenecks. The parameter *f*_*e*_ is not very useful for assessing genetic diversity. First, it only reflects the inequalities in the proportional contributions of founders and does not account for genetic drift [16], and second, it becomes constant after a few generations [19] as the genetic contributions of founders will converge. The parameter *f*_*ge*_ is diminished by both unequal proportional contribution of founders and by genetic drift, that is, discounts *f*_*e*_ to account for the irreversible losses of founder alleles [16]. The value of *f*_*ge*_ represents the cumulative loss of genetic diversity since the base population and directly relates to N_e_ [18,19]. Ratios of the above mentioned parameters of gene origin have been used to interpret the loss in genetic diversity by several authors [23,24,30], but definition and interpretation of the parameter ratios have been diverse and non-quantifiable (in absolute sense).

Genetic diversity measures GD, GD* and GD*-GD were used to quantify the impacts of unequal founder contribution, bottlenecks and random genetic drift. It was estimated that 8.3% of the variation in the base population was lost due to unequal founder contribution (GD* = 0.917) and additional 30.5% due to genetic drift accumulated over non-founder populations (GD*-GD). Consequently, 38.8% of the genetic diversity in the base population was lost from the current living Lundehund population. Proportionally, 78.5% of the total loss was due to random genetic drift and 21.5% due to unequal founder contributions. In fact, the total loss in genetic diversity is simply the average co-ancestry of the chosen reference population [19], which has been between 0.35 and 0.40 since 1980s in Lundehund. During that same time, PCI5 has improved from 0.94 to 1.0, except for the time steps including imported or crossbred individuals. Consequently the *f*_*ge*_ has been constant, as has the total loss in genetic diversity.

Molecular genetic studies have showed extremely low genetic diversity in the Lundehund [2,6,31] and close relatedness between individuals [6]. In a recent study (unpublished data, Stronen A) SNP genotypes were used to estimate heterozygosity in the Lundehund population. The level of heterozygosity was significantly lower (0.038) than in other Nordic Spitz breeds (0.230–0.298). Using this value and our estimate of the level of inbreeding, the heterozygosity prior to the bottlenecks is estimated to be 0.061. This, again, indicates that the Lundehund was already a genetically small population at the time of the start of the pedigree recordings, and that the founder dogs were likely highly related.

### Optimal contribution selection

Very similar results of OCS were obtained from the data sets global, Nordic and Norway, and only the latter is presented here (Table 7). Relaxing the limitation of number of matings allowed per male results in favouring only males least related to the female candidates. In the case of 10 matings allowed per male, only two cross-bred males born in 2014 were selected as sires. If only one mating per male was allowed, the average relatedness of the selected males to the female candidates was 72%, whereas the relatedness between all male candidates to all female candidates was 77%. When 5 or 10 matings per male were allowed, the average relatedness of the selected males to the female candidates was 64% and 38%, respectively. The reduced generation interval with more matings allowed per male, is due to the more intense use of the young crossbred males.

**Table 7:** Optimal contribution selection from the Norwegian Lundehund data, 20 matings requested.

	Maximum number of matings per male		
	1	5	10
Average relationship	0.75	0.73	0.71
Average inbreeding	0.34	0.29	0.21
Generation interval	10.0	8.3	6.2
ΔF	-0.11	-0.22	-0.35
Number of males	20	7	2
Number of females	20	20	20
Maximum avoidance of inbreeding	0.31	0.24	0.21

When the individuals of cross-bred origin (total of 6 individuals) were excluded as breeding candidates, OCS had no effect. The different scenarios of restricted male use resulted in identical levels of average relatedness and inbreeding in the next generation as observed in the last cohort, 76% and 38%, respectively.

The results of the population analysis of Lundehund quantify the narrow genetic base well documented in the breed’s history [1]. Although dog breeders have been very aware of avoiding the mating of close relatives this has had very little effect in the Lundehund population due to the extremely high relatedness among all individuals, which is a result of a low genetic diversity already early in the population history, combined with several severe bottlenecks. This is illustrated by identical levels of expected (under random mating) and observed inbreeding over the past decades.

Due to the extremely high relatedness, OCS alone facilitates no improvement in the current situation in the Lundehund. This was clearly demonstrated by invariant OCS solutions over regions (global, Nordic, Norway). If cross-bred individuals were excluded as breeding candidates, no favourable changes in relatedness were achieved independent on the number of matings allowed per male.

In situations where genetic variation is depleted in a population, migration is the only method to introduce new variation into the population and enable sustainable management and selection [29]. Based on the results of this study, together with previously published results on Lundehund genetic diversity [2,6,31], it is clear that immediate actions are needed to increase the genetic diversity in the current Lundehund population, and that the only option to secure the conservation of this rare breed is to introduce individuals from foreign breeds.

**Fig 3:**
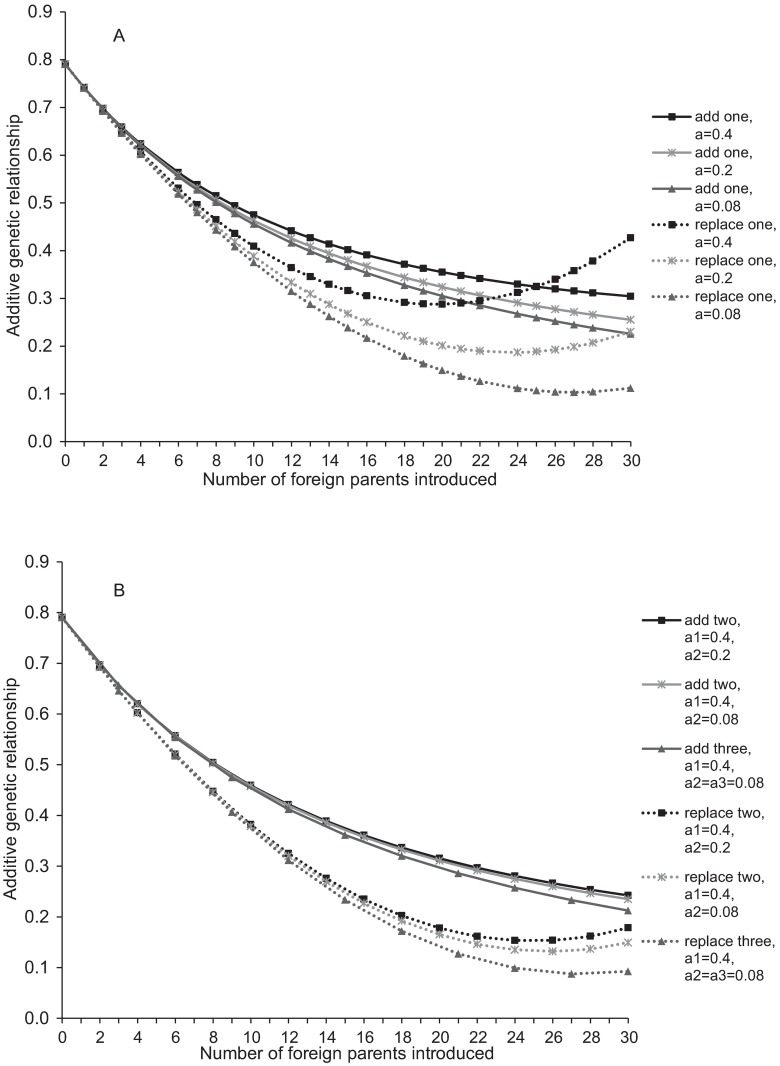
Effect of introduction of foreign breeds into the default Lundehund parental population (N = 30, additive genetic relationship 0.77). Default parental population: N = 30, additive genetic relationship (a) 0.77, inbreeding a/2. (A) Introduction of one breed. (B) Introduction of two or three breeds.

The initialisation of cross-breeding does not offer an immediate remedy to the serious problem of relatedness and inbreeding in the Lundehund population. How much the additive genetic relationship of the breeding candidates, half of the expected F of the progeny, is decreased by the introduction of foreign breeds depends on the number of breeders, number of breeds and individuals of foreign breeds introduced, within-breed additive genetic relationships, and the average inbreeding coefficients of all breeds. For Lundehund (30 purebred breeding candidates with an average additive relationship, *a*, of 0.77), introduction of additional 10 parents of foreign breed reduced the average parental *a* by approximately 40%, down to 0.42–0.48 (Fig 3). This was achieved independently of whether one, two or three breeds (with equal proportions, within breed *a* 0.08–0.4, and F = *a*/2) were introduced. If instead of adding new candidates, ten purebred individuals were replaced by parents from other breeds, a reduction of 48–53% in parental *a* was observed. When breeders of foreign breeds were added to the default Lundehund parental group, the number of breeds and different levels of *a* and F of the foreign populations seemed to have a minor effect on the parental *a*, whereas the number of individuals introduced was decisive (Fig 3). When Lundehund were replaced by foreign breed individuals, larger differences were observed between scenarios of different number of breeds, F and *a* (Fig 3). The effect of cross-breeding on the next generation’s relatedness is conditional on the proportional contributions of foreign breed and purebred parents, which in turn depends on the breeding decisions of private dog owners. The conservation of Lundehund allele variants is difficult to predict as it is conditional on the future use of the cross-bred individuals.

## Conclusions

The Lundehund is an endangered Norwegian dog breed with high recreational and cultural-historical value. Current population is showing signs of inbreeding depression, due to extremely high relatedness of the whole population. Breeding optimisation, including OCS, offer no remedy unless individuals of foreign breed are introduced to the Lundehund. Our data strongly suggests that crossbred individuals should immediately be accepted as part of the main population and as breeding candidates.

## Supporting Information

S1 File Calculation of the additive genetic relationship between breeding candidates when foreign breed(s) are added to the home breed, or the home breed breeders are replaced by foreign breed breeders. (DOCX)
