# Supplementary material for: Cross-Breeding Is Inevitable to Conserve the Highly Inbred Population of Puffin Hunter: The Norwegian Lundehund
Source: PLoS One. 2017 Jan 20;12(1):e0170039. doi: 10.1371/journal.pone.0170039 (PMC5249080; doi:10.1371/journal.pone.0170039)
Supplement: S1 File — (DOCX) [file pone.0170039.s001.docx]

# Non-related breeds:

## ADD foreign breeders:

$$a_{cross}=\frac{N*\left( 1+F \right)+\left( N^{2}-N \right)*a+\sum_{i=1}^{m} \left[ k_{i}*\left( 1+F_{i} \right)+\left( k_{i}^{2}-k_{i} \right)*a_{i} \right]}{\left( N+\sum_{i=1}^{m} k_{i} \right)^{2}}$$

## REPLACE with foreign breeders:

$$a_{cross}=\frac{\left[ \left( N-\sum_{i=1}^{m} k_{i} \right)*\left( 1+F \right) \right]+\left( \left( N-\sum_{i=1}^{m} k_{i} \right)^{2}-\left( N-\sum_{i=1}^{m} k_{i} \right) \right)*a+\sum_{i=1}^{m} \left[ k_{i}*\left( 1+F_{i} \right)+\left( k_{i}^{2}-k_{i} \right)*a_{i} \right]}{N^{2}}$$

a_cross_ = additive genetic relationship between breeders of home and foreign breed(s)

N = number of home breed candidates

F = a/2 = inbreeding in home breed

a = additive genetic relationship between home breed candidates

k_i_ = number of foreign breed candidates, breed i (i=1-m), m=1-3

F_i_ = a_i_/2 = inbreeding in foreign breed i, (i=1-m), m=1-3

a_i_ = additive genetic relationship between foreign breed candidates, breed i, (i=1-m), m=1-3

# Related breeds (only one foreign breed added/replaced):

## ADD foreign breeders:

$$a_{cross}=\frac{N*\left( 1+F \right)+\left( N^{2}-N \right)*a+k*\left( 1+F_{k} \right)+\left( k^{2}-k \right)*a_{k}+2*\left( N*k \right)*a_{HF}}{\left( N+k \right)^{2}}$$

## REPLACE with foreign breeders:

$$a_{cross}=\frac{\left[ \left( N-k \right)*\left( 1+F \right) \right]+\left( \left( N-k \right)^{2}-\left( N-k \right) \right)*a+k*\left( 1+F_{k} \right)+\left( k^{2}-k \right)*a_{k}+2*\left( (N-k)*k \right)*a_{HF}}{N^{2}}$$

a_cross_ = additive genetic relationship between breeders of home and foreign breed(s)

N = number of home breed candidates

F = inbreeding in home breed = a/2

a = additive genetic relationship between home breed candidates

k = number of foreign breed candidates

F_k_ = inbreeding in foreign breed, a_k_/2

a_k_ = additive genetic relationship between foreign breed candidates

a_HF_ = additive genetic relationship between home breed and foreign breed candidates
